# RumimiR: a detailed microRNA database focused on ruminant species

**DOI:** 10.1093/database/baz099

**Published:** 2019-10-14

**Authors:** Céline Bourdon, Philippe Bardou, Etienne Aujean, Sandrine Le Guillou, Gwenola Tosser-Klopp, Fabienne Le Provost

**Affiliations:** 1 Génétique Animale et Biologie Intégrative (GABI), Institut National de la Recherche Agronomique (INRA), AgroParisTech, Université Paris-Saclay, Allée de Vilvert, 78350 Jouy-en-Josas, France; 2 GenPhySE, Université de Toulouse, INRA, Ecole Nationale Vétérinaire de Toulouse (ENVT), 24 Chemin de Borde Rouge, 31320 Castanet-Tolosan, France; 3 Sigenae, INRA, 24 Chemin de Borde Rouge, 31320 Castanet-Tolosan, France

## Abstract

The ever-increasing use of next-generation sequencing technologies to explore the genome has generated large quantities of data in recent years. Numerous publications have described several thousand sequences of microRNAs, all species included. A new database (RumimiR) has been created from the literature to provide a detailed description of microRNAs for three ruminant species: cattle, goats and sheep. To date, 2887, 2733 and 5095 unique microRNAs from bovine, caprine and ovine species, respectively, are included. In addition to the most recent reference genomic position and sequence of each microRNA, this database contains details about the animals, tissue origins and experimental conditions mentioned in the publications. Identity to human or mouse microRNA is also indicated. The RumimiR database allows data filtering by selecting microRNAs on the basis of defined criteria such as animal status or tissue origin. For ruminant studies, RumimiR supplements the widely used miRBase database, by using complementary criteria to allow browsing and filtering, and integrates all newly described published sequences. The principal goal of this database is to provide easy access to all the ruminant microRNAs described in the literature.

## Introduction

MicroRNAs are small, highly conserved, non-coding RNAs ~22 nt in length (1) that participate in the post-transcriptional regulation of genes through their impact on targeted messenger RNAs (mRNAs). This phenomenon can lead to translation repression or degradation of the targeted mRNAs and depends on the base-pair binding of the microRNAs to their target via a recognition site, the `seed’ sequence (2, 3).

Many studies currently use next-generation sequencing (NGS) technology to explore the transcriptome, notably in a context of microRNA discovery. A significant amount of data is thus being generated, which can attain more than 1000 microRNA sequences in a single publication (4, 5). Regularly updated tools for data exploration are therefore required and essential. Animal, plant and virus microRNAs are already listed in several databases, such as miRNEST 2.0 (covering more than 400 different species) (6) or miROrtho, which contains 46 animal genomes (7). Some databases are restricted to human microRNAs in a disease context, such as EpimiRBase (microRNAs associated with epilepsy) (8), miRCancer (microRNAs and cancer) (9) or the MicroRNA SNP Disease Database (MSDD; genetic variants affecting microRNAs in a disease context) (10). The recent miRCarta database (11), which lists microRNAs for 148 species, is solely based on the prediction of novel microRNAs. A database portal, miRToolsGallery, which contains more than 1000 tools for studying, identifying or predicting the targets of microRNAs, has recently been set up (12). The database most widely used at present is miRBase, which was created in 2006 by Griffiths-Jones and collaborators at the University of Manchester (13) and lists microRNAs for 271 species (14).

Studies in cattle, goats and sheep have often focused on production traits such as dairy and meat products (15–17), health [mastitis resistance (18, 19)] or reproduction [fertility and fecundity (20–22)]. Due to genome conservation between the three species, the miRNomes (all microRNAs expressed in a tissue or cell type) are relatively similar. However, there are some differences, as well as those between breeds (23). This explains the importance of generating a database that includes all three species, together with specific information on the breed and physiological status of the animals, as this is frequently lacking in the most commonly employed databases.

RumimiR provides a single access portal to an integrated database containing all the information currently published on ruminant microRNAs. RumimiR is freely available online at the following URL: http://rumimir.sigenae.org/.

This database offers an exhaustive list of bovine, caprine and ovine microRNAs, collected from the literature. Pertinent information, notably in the context of dairy production, has also been added to the description of each mature microRNA, and a filtering option for all these data is included. The entire database (and data filtered by applying one or more filters) can be downloaded in different formats. Moreover, past database versions are traceable and can be downloaded.

**Figure 1:**
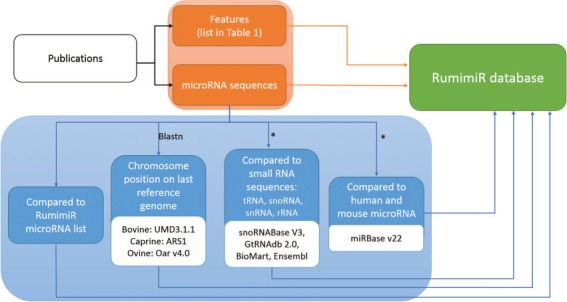
Procedure followed to include microRNAs and their associated features in the RumimiR database. From each publication (black rectangle), the microRNA sequences and their features (orange rectangles) were extracted and included in the RumimiR database. Several analyses were performed to expand the description of the microRNAs collected (blue rectangles): determination of chromosomal position in the latest reference genome of each species (using the NCBI Blastn tool), search for redundancy with microRNAs already present in the RumimiR database, search for identity with other small microRNAs to compare the sequences, search for identity with human or mouse microRNAs present in miRBase. *, using a home-made Python script (Supplementary Figure 2).

## Materials and Methods

### Data collection and processing

RumimiR contains data collected from all publications describing ruminant microRNAs, which corresponds to the 78 publications cited in PubMed (https://www.ncbi.nlm.nih.gov/pubmed). The references for all these publications are listed in Supplementary Data (Supplementary Figure 1). Titles and authors, with a hyperlink to the publication, are provided. RumimiR includes all known microRNA sequences, as well as those described as `novel’ in the publications, which are not always found in the miRBase database. All microRNA sequences were obtained from publications (usually from the text, figures or supplementary data). The microRNA sequences included in RumimiR database are processed and filtered by the authors of each publication. The RumimiR data were standardized by aligning the sequences to a unique reference genome for each species. For this purpose, Blast was implemented using the NCBI tool (24), based on the latest available versions of the reference genome (UMD3.1.1 for bovine, ARS1 for caprine and Oar v4.0 for ovine). The parameters used for the blast analyses were the NCBI Blastn tool (`Somewhat similar sequences’) and general default parameters (short queries; expected threshold, 1000; word size, 7) (24). In the event of multiple alignments, with a whole query cover and 100% identity on the genome, the position of the microRNA sequence could be identified from a Blast of the precursor sequence. Multiple positions were obtained for 344 sequences, even after applying the precursor sequence, when this was available in the publication. These microRNAs have therefore been listed in the RumimiR database without their position, but the number of positions on the genome is shown in the `multi-mapping’ column (*de facto* the chromosome, start and end columns are therefore blank). Characterization of a new microRNA was refined by comparing its sequence with small nucleolar RNA (snoRNA), transfer RNA (tRNA), ribosomal RNA (rRNA) and small nuclear RNA (snRNA) sequences. Indeed, part of the snoRNA structure, the stem loop, is almost identical to that of microRNA (25), and tRNAs with three hairpin loops might also be confused with microRNA. To prevent this, the sequences were therefore compared with all species snoRNA and bovine tRNA present in snoRNABase v3 (26) and GtRNAdb 2.0 (27), respectively, and with the bovine, caprine and ovine rRNA and snRNA sequences present in BioMart, an Ensembl tool (28). When a microRNA displayed 100% identity with a snoRNA, tRNA, rRNA or snRNA over its entire length, it was retained in the RumimiR database but this information is indicated in the `small RNA’ column. A home-made Python script was applied, which compared the microRNA sequences with those extracted from the above-mentioned database (Supplementary Figure 2). Finally, each microRNA was assigned a RumimiR identification number: `Rum-species ID-XXXXX’. The species IDs for cattle, goats and sheep are BTA, CHI or OAR, respectively, and XXXXX is an incremental five-digit number. To add information about a microRNA listed in the RumimiR database, a search was done to determine its identity with human or mouse microRNA. If it had a 100% sequence match with a known microRNA over the full length of the shorter sequence, using the miRBase database (release 22), it was considered identical (14). These identities were verified by applying a Python script to compare the sequences with other microRNA sequences and those extracted from miRBase (Supplementary Figure 2) and to indicate any `strict’ homology with human or mouse microRNA. This step was included because of the large number of publications, and hence details, available for human and mouse microRNAs. All these processes for adding microRNAs to the RumimiR database (Figure 1, blue box) were used to create a four-digit false-positive code representing four positions, each with a value of `0’ or `1’ (0000 to 1111). A `1’ in first position means that the microRNA has multiple genomic locations, and a `1’ in second position means that the microRNA is not already listed in the RumimiR database (a microRNA occurring in different publications is more likely to be a true microRNA). A `1’ in third position means that the microRNA has homology with small RNAs, and a `1’ in fourth position means that the microRNA has no identity with either human or mouse microRNA present in miRBase. One or more values of this four-digit code can be filtered out. A microRNA with a code of 1111 is most likely to be a false positive. This indication enables RumimiR users to select the microRNAs with acceptable false-positive scores.

**Figure 2:**
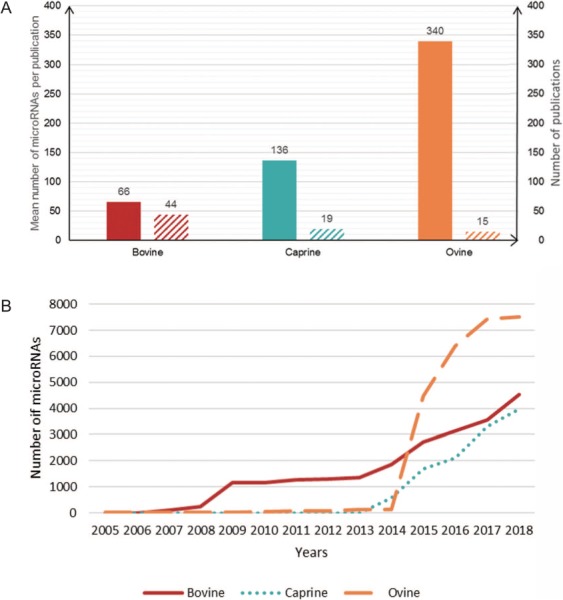
Number of microRNAs per publication. (A) Plain bars, average number of microRNAs described per publication for each species. Hatched bars, the number of publications on each species. (B) Cumulative number of microRNAs described per year in each species.

**Figure 3:**
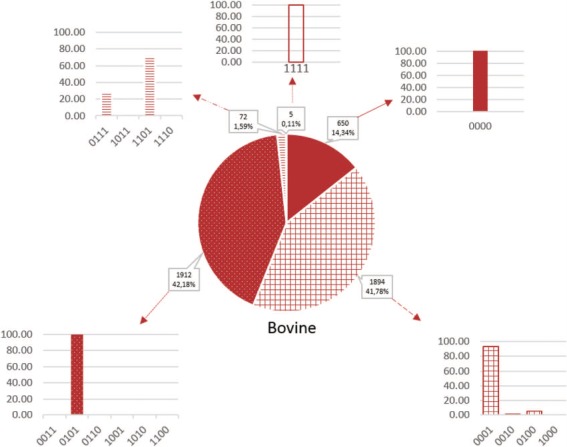
Proportion of microRNAs according to the false-positive codes in cattle. Sequences without a `1’-value are more likely to be true microRNAs and those with 4 1-values are less likely to be microRNAs. Plain color, codes without `1’; gridded color, codes with one `1’; dotted color, codes with two `1’, hatched color, codes with three `1’; empty color, codes with four `1’.

All microRNAs, and most of the features described in the literature, have therefore been documented in the RumimiR database. All the microRNA sequences are noted, as is the presence (or not) of isomiRs (microRNA sequences at the same genomic position, with almost identical sequences). These almost identical sequences are highlighted in a specific column (`isomiRs’) in RumimiR and also if the microRNA sequence belongs to a known microRNA family. The database also contains details of the number of animals studied, their breeds, ages and lactation stage, as well as the tissue of origin, if this was mentioned in the publication.

The RumimiR website was built using HTML5 technology (https://dev.w3.org/html5), the bootstrap front-end framework (https://getbootstrap.com--v4.0.0) with additional jQuery user interface elements (http://jquery.com--v3.2.1) and DataTables jQuery plug-in for the data table (https://datatables.net--v1.10.16), and the plots were implemented by using the Highcharts JavaScript library (http://www.highcharts.com--v6.2.0). RumimiR has been successfully tested on Chrome (version 49 and later) and Firefox (version 57 and later). The data, in JSON format, were provided to the DataTables jQuery plug-in by setting the ajax option to the address of the JSON data source. All statistics, charts and drop-down lists have been built into the data-based fly to make any updates as easy as possible. The Blast tool was developed using Perl-CGI.

The `Release history’ box on the website shows the history of the various RumimiR database versions and allows the user to download entire datasets from current and past versions.

## Results and Discussion

### Content of the RumimiR database

The RumimiR database currently contains 10 715 different microRNAs for 3 ruminant species: 2887 for cattle, 2733 for goats and 5095 for sheep. The average numbers of microRNAs described per publication for bovine, caprine and ovine species are 66, 136 and 340, respectively. The microRNA data were collected from 44 publications for bovines, 19 for caprines and 15 for ovines (Figure 2). The difference in numbers of microRNAs reported for cattle and goats, as compared to sheep, is due to the wider use of NGS technologies in ovine species. More precisely, 16 041 sequences, corresponding to 10 715 different mature microRNAs, are present in the RumimiR database, the difference being due to the presence of isomiRs. A total of 344 microRNA sequences are indicated as having multiple locations (multi-mapping). The microRNAs listed in RumimiR have identities with 889 human or mouse microRNAs: 11.78% of the described microRNAs show sequence identities with both human and mouse microRNAs and 4.66% present sequence identity with only 1 human or 1 mouse microRNA. Comparisons with snoRNA, tRNA, rRNA and snRNA sequences revealed that 48 microRNAs displayed sequence identities with part of a tRNA, 47 with part of a snoRNA, 34 with part of an rRNA and 9 with part of an snRNA.

The importance of each microRNA is evaluated from the false-positive score. Around 50% of the microRNAs (56.1% in bovine, 51.2% in caprine and 49.8% in ovine species) have a score with a low risk of being false positive (code containing 0 or 1 `1’). Moreover, almost every microRNA has a code containing 0–2 `1’ (98.3% in bovine, 92.8% in caprine and 99.4% in ovine species; Figure 3 and Supplementary Figure 3). Each score is represented with the corresponding encoding: for example, the scores of 1 are due to the absence of identity with a mouse and human microRNA, in the three species. In fact, when other elements appeared (i.e. small RNA or multi-mapping), they are often associated with another false positivity element, as seen in Figure 3.

**Figure 4:**
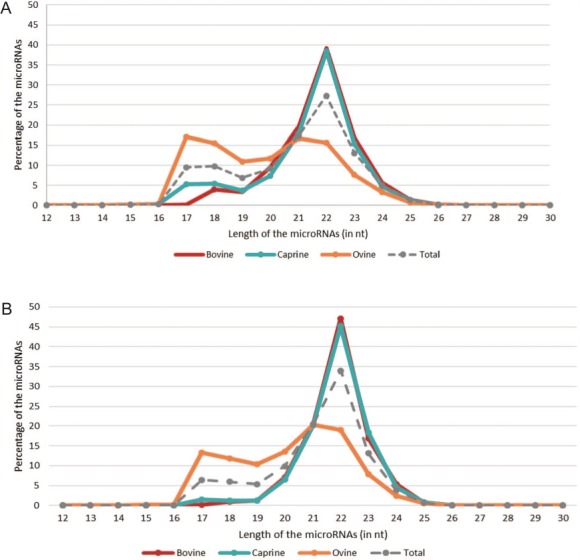
microRNA length distributions across species. The red, blue and orange lines correspond to the percentages of bovine, caprine and ovine microRNAs, respectively, based on their length [in nucleotides (nt)]. The gray dotted line represents all the microRNAs listed in RumimiR. (A) Percentage of all sequences collected in RumimiR. (B) Percentage of sequences with a low false-positive score (code containing 0 and 1 `1’).

Most of the microRNAs listed in RumimiR have the expected length (20–24 nt) (1), with 99.27% having 17–25 nt. The microRNA length distributions in bovine and caprine species are similar (Figure 4A) with 91% of bovines and 84% in caprines having 20–24 nt. The length distribution in ovine species is unusual (55% having 20–24 nt). The numbers of microRNAs with lengths of 17–18 nt and those with 21–22 nt are similar. The proportion differs if only those microRNAs with weak false-positive score (code containing 0 and 1 `1’) are considered, but the percentage of microRNAs with lengths of 17–18 nt is still high (~25%; Figure 4B). Most of the ovine sequences with lengths of 17 and 18 nt were obtained from a single paper (4). The authors explained, in their discussion, that they had discovered a large number of new microRNAs because they had not applied the commonly used restricting pre-screening. All sequences detected at a low-count level or with a low-sample frequency were considered. Most of the ovine sequences with lengths of 17 and 18 nt correspond to new microRNAs.

**Figure 5:**
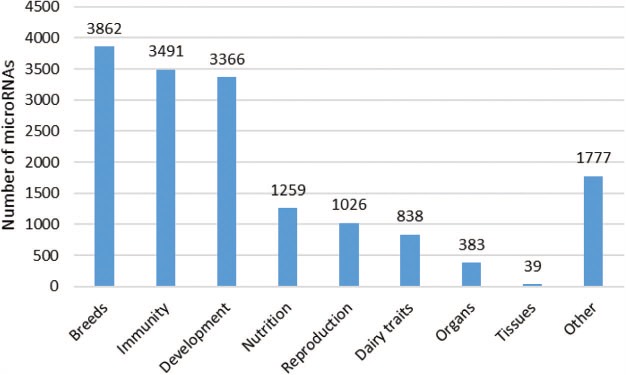
Number of microRNAs according to the conditions studied. The different topics presented here are in line with the issues addressed in the publications. A total of 78 publications were considered, and each article is mentioned only once. Each microRNA was counted several times, if they were described in several publications corresponding to different topics.

The majority of microRNAs in the database were taken from studies based on differences between breeds, developmental stages (mainly in a context of meat production) or immune response (comparison of healthy animals with those suffering from mastitis, a widely-studied pathology in ruminants; Figure 5). The different issues addressed in the publications were implemented as filtering options in the RumimiR database. Thus, 40 different breeds (17 bovine, 14 caprine and 9 ovine breeds, both meat and dairy) are represented along with about 30 tissues and body fluids such as milk (29, 30), adipose tissue (4), mammary gland (31–33) and ovaries (22, 34, 35). Around half of the microRNAs described in the RumimiR database are associated with a single tissue (these `specific’ microRNAs are highlighted by the symbol `⊙’). While the probability of a microRNA being a false positive decreases if it is described in several studies, some of the microRNAs described only once can be specific to a breed, tissue, physiological stage or particular condition.

**Figure 6:**
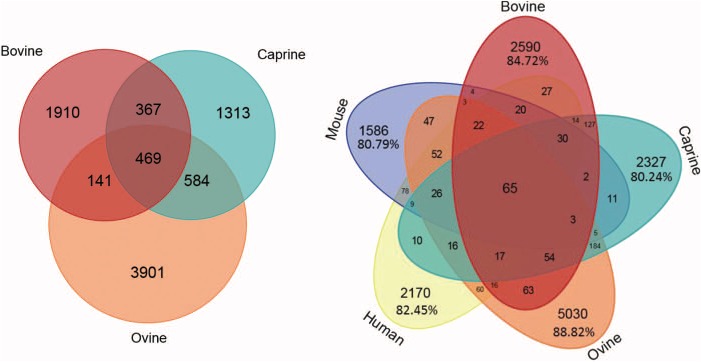
Number of microRNAs common to different species. Venn diagram showing bovine, caprine and ovine unique microRNAs listed in the RumimiR database. Venn diagram showing bovine, caprine and ovine microRNAs listed in the RumimiR database together with the human and mouse microRNAs listed in miRBase (release 22).

Age was mentioned in 38% of the publications, and more than 30 different ages have been listed. Some studies described the differential expression of microRNAs throughout development, and at different ages, which is why several ages might be considered in a single publication. The same was true of lactation stages. All these figures will increase as the database is updated. The numbers of microRNAs common to all three ruminant species, or sequences common to these species and to human and mouse sequences, or specific to each species, are presented in Figure 6. Between 80.24% and 88.82% are species-specific microRNAs.

RumimiR is limited to three ruminant species, unlike miRBase, which is the most widely used and complete database in terms of the number of species covered (plants, animals and viruses). However, this restriction enabled us to generate a more detailed database containing all the microRNAs described in different publications as well as numerous features. Six times more microRNAs are listed in the RumimiR database for bovine, caprine and ovine species than in miRBase: 10 715 in RumimiR versus 1614 in miRBase. Although the latest miRBase release (v22) is quite recent (October 2018), it included only 5% of the data available on deep sequencing of small RNA (14). The risk of assembling all microRNAs mentioned in the literature is still to include false-positive microRNAs, which is why this risk has been evaluated for each sequence. Thus, RumimiR offers a complete and more precise microRNA database for cattle, goats and sheep and should therefore be of value to scientists working on livestock species.

The RumimiR database is extensive because it lists all the features mentioned in the publications (unlike miRBase and other databases), the data are homogenized (with blast on the same reference genomes for example), and because information is included about the identity, small RNAs etc. In addition, the RumimiR database combines the results of a multitude of studies in a single location.

### User interface

**Figure 7:**
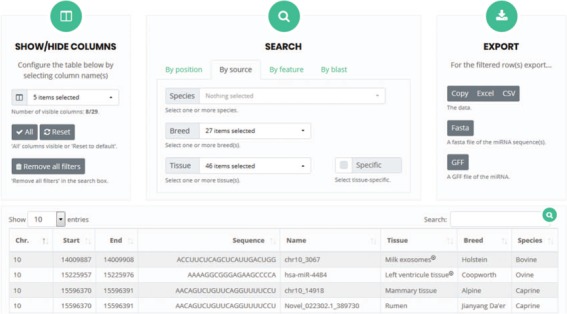
User interface for `search and browse’. Columns can be selected so that only specific features will be visible. The search box allows the detection of microRNAs present in the RumimiR database as a function of the selected choices. The results can be exported in different formats, such as Excel or Fasta.

The web interface is user friendly, allows visualization of all the microRNAs described in bovine, caprine and ovine species and provides at minimum the genomic position (chromosome, start and end) of each microRNA. Some or all the columns can be selected to obtain the sequence, name, tissue or other features of the study and of the microRNAs, by clicking on the appropriate name in the `show/hide columns’ box (Figure 7).

The features available for the various microRNAs, such as the sequence, related publications, name, isomiRs, family, tissue origin, breed, condition studied and others are listed in the 29 columns shown in Table 1.

**Table 1:** List of columns presenting the microRNAs and features detailed in the RumimiR database

**Column name**	**Description**
Chr (Start, End)	Genomic position of the microRNA
Multi-mapping	Number of localizations in case of multi-mapping (with 100% query cover and 100% identity)
Sequence	Sequence of the mature microRNA
Publication	Publication in which the microRNA is described
Name	Name assigned to the microRNA
IsomiRs	Name of isomiRs of the microRNA (sequence and genomic position almost identical)
RumimiR ID	Name of the microRNA with the RumimiR nomenclature
Family	microRNA family to which the microRNA is affiliated
Tissue	Tissue in which the microRNA was discovered
Breed	Breed used during the study
Study conditions	Conditions prevailing during the study
Lactation stages	Lactation stages of the animals studied
Age	Age of the animals studied
Species	Species of the animals studied (bovine, caprine or ovine)
Number	Number of novel microRNAs described in the publication
Cut-off	Cut-off point used in the publication to detect novel microRNAs
Method	Method used to detect novel microRNAs
Number and breed	Number of animals studied in each breed
Reference genome	UMD3.1.1, ARS1 or Oar v4.0
Bioinformatics tools used	Software used in the study for the microRNA detection
Condition details	Details of study conditions (i.e. number of animals per condition)
5p/3p	If the microRNA is 5p or 3p
Strand (+/−)	Strand in which the microRNA is situated
Star sequence	Sequence of the star microRNA
Matching seed	Known microRNA with the same seed
small RNAs	If the microRNA described is in fact part of a snoRNA, tRNA, snRNA or rRNA
hsa homology	Similarity with a human microRNA
mmu homology	Similarity with a mouse microRNA
False-positive code	Four-digit false-positive code

Gray lines indicate a feature that is present in RumimiR but not in miRBase.

Users can also visualize those microRNAs of solely personal interest by selecting the corresponding column (sequence and/or tissue and/or breed etc.) and then using the `search’ box to define different options such as `by position’ (chromosome, start and end), `by source’ (species, breed and tissue) or `by feature’ (conditions, method and software; Figure 7). The above-mentioned criteria can also be applied to filter all the mature microRNAs listed. A sequence or key word can also be entered in the `Search’ box to extract a list of the relevant data in the results table. The name of each microRNA in the `name’ column is the one given in the original publication. If specific requests are selected, the appropriate filtered data appear and can be downloaded in several formats (Excel, CSV, Fasta or GFF). Users can thus obtain all the data that they require, appropriately filtered and rapidly. They can also download the entire database without applying any filters.

For clarity, some statistics and graphs, which summarize the data presented in the database (Supplementary Figure 4), are provided in the online version. These include, among others, the date of the latest update, the number of sequences listed and the number of publications involved. The distribution of microRNAs by species and by breed is presented graphically, as is the distribution by tissue origin.

RumimiR also includes an alignment tool so that sequences can be submitted for analysis and the corresponding microRNAs sorted according to the resulting hits.

### Future extensions

The NGS approach is generating extensive data, and the number of microRNAs will continue to rise in the near future. These new data will be integrated and the database regularly updated (at least twice a year) so that it remains exhaustive. Identity with human or mouse microRNAs will be checked each time that a new release of miRBase becomes available. The three ruminant species probably contain a comparable number of microRNAs as the difference between the numbers of bovine (2887), caprine (2733) and ovine (5095) microRNAs could be due to the more intense efforts assigned to ovine sequencing. However, the numbers of human or mouse microRNAs in the latest release of miRBase are 2654 and 1978, respectively. MicroRNA detection in ruminant species, as compared to humans and mice, may have reached saturation. In addition, the numbers will probably evolve as the genome assemblies are updated and the same quality of contiguity and annotation is attained as for human and mouse species.

One potential extension for RumimiR might be to add genetic variants of ruminant microRNAs (`miRSNPs’ for microRNA and single nucleotide polymorphisms). First, those linked with dairy QTL, and then those associated with health or meat QTL. Publications on these genetic microRNA variations are indeed increasingly numerous. For example, in humans, the MSDD database has been created exclusively for miRSNPs linked to human diseases (10). In the same way, the RumimiR database could be completed by including ruminant miRSNPs. Other features and filters will be added depending on the features described in the literature and the needs of the scientific community. Evolution of the reference genomes will also be considered, to take into account the genomic positions of microRNAs in the latest versions of the reference genomes. The RumimiR database could also be extended to include other livestock species.

## Conclusion

The RumimiR database contains an exhaustive list of all microRNAs described in publications pertaining to three livestock species: cattle, goats and sheep. This database supplements miRBase, one of the most widely used microRNA databases, by including various features mentioned in the literature, which are important in the context of animal production and dairy traits, notably the breeds of animals studied or the tissues in which the microRNAs were described. RumimiR can be used to retrieve specific microRNAs and thus provide additional information about the livestock species and a clearer understanding of the context in which these microRNAs were discovered. The database will be regularly updated and will continue to be exhaustive. RumimiR, by standardizing and centralizing information from a large number of publications, constitutes a unique tool that presents and describes all known microRNAs in ruminants.

## Supplementary Material

Supp_Figure_1_baz099

Supp_Figure_2_baz099

Supp_Figure_3_baz099

Supp_Figure_4_baz099
