# Supplementary material for: RumimiR: a detailed microRNA database focused on ruminant species
Source: Database (Oxford). 2019 Oct 14;2019:baz099. doi: 10.1093/database/baz099 (PMC6790497; doi:10.1093/database/baz099)
Supplement: Supp_Figure_1_baz099 [file supp_figure_1_baz099.docx]

1. Al-Husseini,W., Chen,Y., Gondro,C., Herd,R.M., Gibson,J.P. and Arthur,P.F. (2015) Characterization and Profiling of Liver microRNAs by RNA-sequencing in Cattle Divergently Selected for Residual Feed Intake. *Asian-Australas. J. Anim. Sci.*, **29**, 1371–1382.

2. Artzi,S., Kiezun,A. and Shomron,N. (2008) miRNAminer: A tool for homologous microRNA gene search. *BMC Bioinformatics*, **9**, 39.

3. Bu,D.P., Nan,X.M., Wang,F., Loor,J.J. and Wang,J.Q. (2015) Identification and characterization of microRNA sequences from bovine mammary epithelial cells. *J. Dairy Sci.*, **98**, 1696–1705.

4. Coutinho,L.L., Matukumalli,L.K., Sonstegard,T.S., Van Tassell,C.P., Gasbarre,L.C., Capuco,A.V. and Smith,T.P.L. (2007) Discovery and profiling of bovine microRNAs from immune-related and embryonic tissues. *Physiol. Genomics*, **29**, 35–43.

5. Do,D.N., Li,R., Dudemaine,P.-L. and Ibeagha-Awemu,E.M. (2017) MicroRNA roles in signalling during lactation: an insight from differential expression, time course and pathway analyses of deep sequence data. *Sci. Rep.*, **7**, 44605.

6. Fang,L., Hou,Y., An,J., Li,B., Song,M., Wang,X., Sørensen,P., Dong,Y., Liu,C., Wang,Y., *et al.* (2016) Genome-Wide Transcriptional and Post-transcriptional Regulation of Innate Immune and Defense Responses of Bovine Mammary Gland to Staphylococcus aureus. *Front. Cell. Infect. Microbiol.*, **6**.

7. Farrell,D., Shaughnessy,R.G., Britton,L., MacHugh,D.E., Markey,B. and Gordon,S.V. (2015) The Identification of Circulating MiRNA in Bovine Serum and Their Potential as Novel Biomarkers of Early Mycobacterium avium subsp paratuberculosis Infection. *PLOS ONE*, **10**, e0134310.

8. Gebremedhn,S., Salilew-Wondim,D., Ahmad,I., Sahadevan,S., Hossain,M.M., Hoelker,M., Rings,F., Neuhoff,C., Tholen,E., Looft,C., *et al.* (2015) MicroRNA Expression Profile in Bovine Granulosa Cells of Preovulatory Dominant and Subordinate Follicles during the Late Follicular Phase of the Estrous Cycle. *PLOS ONE*, **10**, e0125912.

9. Glazov,E.A., Kongsuwan,K., Assavalapsakul,W., Horwood,P.F., Mitter,N. and Mahony,T.J. (2009) Repertoire of Bovine miRNA and miRNA-Like Small Regulatory RNAs Expressed upon Viral Infection. *PLoS ONE*, **4**, e6349.

10. Guduric-Fuchs,J., O’Connor,A., Cullen,A., Harwood,L., Medina,R.J., O’Neill,C.L., Stitt,A.W., Curtis,T.M. and Simpson,D.A. (2012) Deep sequencing reveals predominant expression of miR-21 amongst the small non-coding RNAs in retinal microvascular endothelial cells. *J. Cell. Biochem.*, **113**, 2098–2111.

11. Hossain,M., Ghanem,N., Hoelker,M., Rings,F., Phatsara,C., Tholen,E., Schellander,K. and Tesfaye,D. (2009) Identification and characterization of miRNAs expressed in the bovine ovary. *BMC Genomics*, **10**, 443.

12. Huang,J., Ju,Z., Li,Q., Hou,Q., Wang,C., Li,J., Li,R., Wang,L., Sun,T., Hang,S., *et al.* (2011) Solexa sequencing of novel and differentially expressed microRNAs in testicular and ovarian tissues in Holstein cattle. *Int. J. Biol. Sci.*, **7**, 1016–1026.

13. Jin,W., Dodson,M.V., Moore,S.S., Basarab,J.A. and Guan,L.L. (2010) Characterization of microRNA expression in bovine adipose tissues: a potential regulatory mechanism of subcutaneous adipose tissue development. *BMC Mol. Biol.*, **11**, 29.

14. Jin,W., Grant,J.R., Stothard,P., Moore,S.S. and Guan,L. (2009) Characterization of bovine miRNAs by sequencing and bioinformatics analysis. *BMC Mol. Biol.*, **10**, 90.

15. Jin,W., Ibeagha-Awemu,E.M., Liang,G., Beaudoin,F., Zhao,X. and Guan,L. (2014) Transcriptome microRNA profiling of bovine mammary epithelial cells challenged with Escherichia coli or Staphylococcus aureus bacteria reveals pathogen directed microRNA expression profiles. *BMC Genomics*, **15**, 181.

16. Ju,Z., Jiang,Q., Liu,G., Wang,X., Luo,G., Zhang,Y., Zhang,J., Zhong,J. and Huang,J. (2018) Solexa sequencing and custom microRNA chip reveal repertoire of microRNAs in mammary gland of bovine suffering from natural infectious mastitis. *Anim. Genet.*, **49**, 3–18.

17. Lawless,N., Foroushani,A.B.K., McCabe,M.S., O’Farrelly,C. and Lynn,D.J. (2013) Next Generation Sequencing Reveals the Expression of a Unique miRNA Profile in Response to a Gram-Positive Bacterial Infection. *PLoS ONE*, **8**, e57543.

18. Lawless,N., Reinhardt,T.A., Bryan,K., Baker,M., Pesch,B., Zimmerman,D., Zuelke,K., Sonstegard,T., O’Farrelly,C., Lippolis,J.D., *et al.* (2014) MicroRNA Regulation of Bovine Monocyte Inflammatory and Metabolic Networks in an *In Vivo* Infection Model. *G3amp58 GenesGenomesGenetics*, **4**, 957–971.

19. Le Guillou,S., Marthey,S., Laloë,D., Laubier,J., Mobuchon,L., Leroux,C. and Le Provost,F. (2014) Characterisation and Comparison of Lactating Mouse and Bovine Mammary Gland miRNomes. *PLoS ONE*, **9**, e91938.

20. Li,J., Mao,L., Li,W., Hao,F., Zhong,C., Zhu,X., Ji,X., Yang,L., Zhang,W., Liu,M., *et al.* (2018) Analysis of microRNAs Expression Profiles in Madin-Darby Bovine Kidney Cells Infected With Caprine Parainfluenza Virus Type 3. *Front. Cell. Infect. Microbiol.*, **8**.

21. Li,R., Beaudoin,F., Ammah,A.A., Bissonnette,N., Benchaar,C., Zhao,X., Lei,C. and Ibeagha-Awemu,E.M. (2015) Deep sequencing shows microRNA involvement in bovine mammary gland adaptation to diets supplemented with linseed oil or safflower oil. *BMC Genomics*, **16**.

22. Li,R., Dudemaine,P.-L., Zhao,X., Lei,C. and Ibeagha-Awemu,E.M. (2016) Comparative Analysis of the miRNome of Bovine Milk Fat, Whey and Cells. *PLOS ONE*, **11**, e0154129.

23. Liang,G., Malmuthuge,N., McFadden,T.B., Bao,H., Griebel,P.J., Stothard,P. and Guan,L.L. (2014) Potential Regulatory Role of MicroRNAs in the Development of Bovine Gastrointestinal Tract during Early Life. *PLoS ONE*, **9**, e92592.

24. Long,J.-E. and Chen,H.-X. (2009) Identification and Characteristics of Cattle MicroRNAs by Homology Searching and Small RNA Cloning. *Biochem. Genet.*, **47**, 329–343.

25. Lv,Y., Wang,Y., Sun,J., Gong,C., Chen,Y., Su,G., Gao,G., Bai,C., Wei,Z., Zhang,L., *et al.* (2017) MicroRNA profiles of fibroblasts derived from in vivo fertilized and fat-1 transgenic cattle. *Gene*, **636**, 70–77.

26. Maalouf,S.W., Liu,W.-S., Albert,I. and Pate,J.L. (2014) Regulating life or death: Potential role of microRNA in rescue of the corpus luteum. *Mol. Cell. Endocrinol.*, **398**, 78–88.

27. Malvisi,M., Palazzo,F., Morandi,N., Lazzari,B., Williams,J.L., Pagnacco,G. and Minozzi,G. (2016) Responses of Bovine Innate Immunity to Mycobacterium avium subsp. paratuberculosis Infection Revealed by Changes in Gene Expression and Levels of MicroRNA. *PLOS ONE*, **11**, e0164461.

28. Muroya,S., Taniguchi,M., Shibata,M., Oe,M., Ojima,K., Nakajima,I. and Chikuni,K. (2013) Profiling of differentially expressed microRNA and the bioinformatic target gene analyses in bovine fast- and slow-type muscles by massively parallel sequencing1. *J. Anim. Sci.*, **91**, 90–103.

29. Salilew-Wondim,D., Ahmad,I., Gebremedhn,S., Sahadevan,S., Hossain,M.M., Rings,F., Hoelker,M., Tholen,E., Neuhoff,C., Looft,C., *et al.* (2014) The Expression Pattern of microRNAs in Granulosa Cells of Subordinate and Dominant Follicles during the Early Luteal Phase of the Bovine Estrous Cycle. *PLoS ONE*, **9**, e106795.

30. Shen,B., Zhang,L., Lian,C., Lu,C., Zhang,Y., Pan,Q., Yang,R. and Zhao,Z. (2016) Deep Sequencing and Screening of Differentially Expressed MicroRNAs Related to Milk Fat Metabolism in Bovine Primary Mammary Epithelial Cells. *Int. J. Mol. Sci.*, **17**, 200.

31. Singh,J., Mukhopadhyay,C.S., Kaur,S., Malhotra,P., Sethi,R.S. and Choudhary,R.K. (2016) Identification of the MicroRNA Repertoire in TLR-Ligand Challenged Bubaline PBMCs as a Model of Bacterial and Viral Infection. *PLOS ONE*, **11**, e0156598.

32. Strozzi,F., Mazza,R., Malinverni,R. and Williams,J.L. (2009) Annotation of 390 bovine miRNA genes by sequence similarity with other species. *Anim. Genet.*, **40**, 125–125.

33. Sun,J., Sonstegard,T.S., Li,C., Huang,Y., Li,Z., Lan,X., Zhang,C., Lei,C., Zhao,X. and Chen,H. (2015) Altered microRNA expression in bovine skeletal muscle with age. *Anim. Genet.*, **46**, 227–238.

34. Sun,J., Aswath,K., Schroeder,S.G., Lippolis,J.D., Reinhardt,T.A. and Sonstegard,T.S. (2015) MicroRNA expression profiles of bovine milk exosomes in response to Staphylococcus aureus infection. *BMC Genomics*, **16**.

35. Sun,J., Zhang,B., Lan,X., Zhang,C., Lei,C. and Chen,H. (2014) Comparative Transcriptome Analysis Reveals Significant Differences in MicroRNA Expression and Their Target Genes between Adipose and Muscular Tissues in Cattle. *PLoS ONE*, **9**, e102142.

36. Sun,J., Zhou,Y., Cai,H., Lan,X., Lei,C., Zhao,X., Zhang,C. and Chen,H. (2014) Discovery of Novel and Differentially Expressed MicroRNAs between Fetal and Adult Backfat in Cattle. *PLoS ONE*, **9**, e90244.

37. Tesfaye,D., Worku,D., Rings,F., Phatsara,C., Tholen,E., Schellander,K. and Hoelker,M. (2009) Identification and expression profiling of microRNAs during bovine oocyte maturation using heterologous approach. *Mol. Reprod. Dev.*, **76**, 665–677.

38. Vegh,P., Foroushani,A.B.K., Magee,D.A., McCabe,M.S., Browne,J.A., Nalpas,N.C., Conlon,K.M., Gordon,S.V., Bradley,D.G., MacHugh,D.E., *et al.* (2013) Profiling microRNA expression in bovine alveolar macrophages using RNA-seq. *Vet. Immunol. Immunopathol.*, **155**, 238–244.

39. Vegh,P., Magee,D.A., Nalpas,N.C., Bryan,K., McCabe,M.S., Browne,J.A., Conlon,K.M., Gordon,S.V., Bradley,D.G., MacHugh,D.E., *et al.* (2015) MicroRNA profiling of the bovine alveolar macrophage response to Mycobacterium bovis infection suggests pathogen survival is enhanced by microRNA regulation of endocytosis and lysosome trafficking. *Tuberculosis*, **95**, 60–67.

40. Wang,H., Xiao,S., Wang,M., Kim,N.-H., Li,H. and Wang,G. (2015) In silico identification of conserved microRNAs and their targets in bovine fat tissue. *Gene*, **559**, 119–128.

41. Wu,H., Zhang,T., Ma,X., Jiang,K., Zhao,G., Qiu,C. and Deng,G. (2017) Specific microRNA library of IFN-T on bovine endometrial epithelial cells. *Oncotarget*, **8**.

42. Xu,C., Wu,S., Zhao,W., Mipam,T., Liu,J., Liu,W., Yi,C., Shah,M. ali, Yu,S. and Cai,X. (2018) Differentially expressed microRNAs between cattleyak and yak testis. *Sci. Rep.*, **8**.

43. Xu,Y., Niu,J., Xi,G., Niu,X., Wang,Y., Guo,M., Yangzong,Q., Yao,Y., Sizhu,S.L. and Tian,J. (2018) TGF-β1 resulting in differential microRNA expression in bovine granulosa cells. *Gene*, **663**, 88–100.

44. Zhang,W.W., Sun,X.F., Tong,H.L., Wang,Y.H., Li,S.F., Yan,Y.Q. and Li,G.P. (2016) Effect of differentiation on microRNA expression in bovine skeletal muscle satellite cells by deep sequencing. *Cell. Mol. Biol. Lett.*, **21**.

45. An,X., Song,Y., Hou,J., Li,G., Zhao,H., Wang,J. and Cao,B. (2016) Identification and profiling of microRNAs in the ovaries of polytocous and monotocous goats during estrus. *Theriogenology*, **85**, 769–780.

46. Capra,E., Lazzari,B., Frattini,S., Chessa,S., Coizet,B., Talenti,A., Castiglioni,B., Marsan,P.A., Crepaldi,P., Pagnacco,G., *et al.* (2018) Distribution of ncRNAs expression across hypothalamic-pituitary-gonadal axis in Capra hircus. *BMC Genomics*, **19**.

47. Guo,J., Zhao,W., Zhan,S., Li,L., Zhong,T., Wang,L., Dong,Y. and Zhang,H. (2016) Identification and Expression Profiling of miRNAome in Goat longissimus dorsi Muscle from Prenatal Stages to a Neonatal Stage. *PLOS ONE*, **11**, e0165764.

48. Hou,J., An,X., Song,Y., Cao,B., Yang,H., Zhang,Z., Shen,W. and Li,Y. (2017) Detection and comparison of microRNAs in the caprine mammary gland tissues of colostrum and common milk stages. *BMC Genet.*, **18**.

49. Ling,Y.H., Guo,X.F., Chen,T., Ding,J.P., Ma,Y.H., Chu,M.X., Di,R., Zhang,Y.H. and Zhang,X.R. (2016) Characterization and analysis of differentially expressed microRNAs in hircine ovaries during the follicular and luteal phases. *Anim. Reprod. Sci.*, **166**, 47–57.

50. Ling,Y.-H., Ren,C.-H., Guo,X.-F., Xu,L.-N., Huang,Y.-F., Luo,J.-C., Zhang,Y.-H., Zhang,X.-R. and Zhang,Z.-J. (2014) Identification and characterization of microRNAs in the ovaries of multiple and uniparous goats (Capra hircus) during follicular phase. *BMC Genomics*, **15**, 339.

51. Mobuchon,L., Marthey,S., Le Guillou,S., Laloë,D., Le Provost,F. and Leroux,C. (2015) Food Deprivation Affects the miRNome in the Lactating Goat Mammary Gland. *PLOS ONE*, **10**, e0140111.

52. Qi,X., Wang,T., Xue,Q., Li,Z., Yang,B. and Wang,J. (2018) MicroRNA expression profiling of goat peripheral blood mononuclear cells in response to peste des petits ruminants virus infection. *Vet. Res.*, **49**.

53. Qu,B., Qiu,Y., Zhen,Z., Zhao,F., Wang,C., Cui,Y., Li,Q. and Zhang,L. (2016) Computational identification and characterization of novel microRNA in the mammary gland of dairy goat (Capra hircus). *J. Genet.*, **95**, 625–637.

54. Song,Y., An,X., Zhang,L., Fu,M., Peng,J., Han,P., Hou,J., Zhou,Z. and Cao,B. (2015) Identification and Profiling of microRNAs in Goat Endometrium during Embryo Implantation. *PLOS ONE*, **10**, e0122202.

55. Su,R., Fu,S., Zhang,Y., Wang,R., Zhou,Y., Li,J. and Zhang,W. (2015) Comparative genomic approach reveals novel conserved microRNAs in Inner Mongolia cashmere goat skin and longissimus dorsi. *Mol. Biol. Rep.*, **42**, 989–995.

56. Wang,S., Ge,W., Luo,Z., Guo,Y., Jiao,B., Qu,L., Zhang,Z. and Wang,X. (2017) Integrated analysis of coding genes and non-coding RNAs during hair follicle cycle of cashmere goat (Capra hircus). *BMC Genomics*, **18**.

57. Wang,Y., Zhang,C., Fang,X., Zhao,Y., Chen,X., Sun,J., Zhou,Y., Wang,J., Wang,Y., Lan,X., *et al.* (2014) Identification and Profiling of microRNAs and Their Target Genes from Developing Caprine Skeletal Muscle. *PLoS ONE*, **9**, e96857.

58. Wu,J., Zhu,H., Song,W., Li,M., Liu,C., Li,N., Tang,F., Mu,H., Liao,M., Li,X., *et al.* (2014) Identification of Conservative MicroRNAs in Saanen Dairy Goat Testis Through Deep Sequencing. *Reprod. Domest. Anim.*, **49**, 32–40.

59. Wu,Z., Fu,Y., Cao,J., Yu,M., Tang,X. and Zhao,S. (2014) Identification of Differentially Expressed miRNAs between White and Black Hair Follicles by RNA-Sequencing in the Goat (Capra hircus). *Int. J. Mol. Sci.*, **15**, 9531–9545.

60. Ye,J., Yao,Z., Si,W., Gao,X., Yang,C., Liu,Y., Ding,J., Huang,W., Fang,F. and Zhou,J. (2018) Identification and characterization of microRNAs in the pituitary of pubescent goats. *Reprod. Biol. Endocrinol.*, **16**.

61. Zhong,T., Hu,J., Xiao,P., Zhan,S., Wang,L., Guo,J., Li,L., Zhang,H. and Niu,L. (2017) Identification and Characterization of MicroRNAs in the Goat (Capra hircus) Rumen during Embryonic Development. *Front. Genet.*, **8**.

62. Zhu,L., Chen,T., Sui,M., Han,C., Fang,F., Ma,Y., Chu,M., Zhang,X., Liu,C. and Ling,Y. (2016) Comparative profiling of differentially expressed microRNAs between the follicular and luteal phases ovaries of goats. *SpringerPlus*, **5**.

63. Zi,X., Lu,J. and Ma,L. (2017) Identification and comparative analysis of the ovarian microRNAs of prolific and non-prolific goats during the follicular phase using high-throughput sequencing. *Sci. Rep.*, **7**.

64. Caiment,F., Charlier,C., Hadfield,T., Cockett,N., Georges,M. and Baurain,D. (2010) Assessing the effect of the CLPG mutation on the microRNA catalog of skeletal muscle using high-throughput sequencing. *Genome Res.*, **20**, 1651–1662.

65. Chang,W., Wang,J., Tao,D., Zhang,Y., He,J. and Shi,C. (2015) Identification of a novel miRNA from the ovine ovary by a combinatorial approach of bioinformatics and experiments. *J. Vet. Med. Sci.*, **77**, 1617–1624.

66. Davis,E., Caiment,F., Tordoir,X., Cavaillé,J., Ferguson-Smith,A., Cockett,N., Georges,M. and Charlier,C. (2005) RNAi-Mediated Allelic trans-Interaction at the Imprinted Rtl1/Peg11 Locus. *Curr. Biol.*, **15**, 743–749.

67. Du,J., Gao,S., Tian,Z., Xing,S., Huang,D., Zhang,G., Zheng,Y., Liu,G., Luo,J., Chang,H., *et al.* (2017) MicroRNA expression profiling of primary sheep testicular cells in response to bluetongue virus infection. *Infect. Genet. Evol.*, **49**, 256–267.

68. Galio,L., Droineau,S., Yeboah,P., Boudiaf,H., Bouet,S., Truchet,S. and Devinoy,E. (2013) MicroRNA in the ovine mammary gland during early pregnancy: spatial and temporal expression of miR-21, miR-205, and miR-200. *Physiol. Genomics*, **45**, 151–161.

69. Hu,X., Pokharel,K., Peippo,J., Ghanem,N., Zhaboyev,I., Kantanen,J. and Li,M.-H. (2016) Identification and characterization of miRNAs in the ovaries of a highly prolific sheep breed. *Anim. Genet.*, **47**, 234–239.

70. Laganà,A., Veneziano,D., Spata,T., Tang,R., Zhu,H., Mohler,P.J. and Kilic,A. (2015) Identification of General and Heart-Specific miRNAs in Sheep (Ovis aries). *PLOS ONE*, **10**, e0143313.

71. Liu,Y., Zhang,J., Xu,Q., Kang,X., Wang,K., Wu,K. and Fang,M. (2018) Integrated miRNA-mRNA analysis reveals regulatory pathways underlying the curly fleece trait in Chinese tan sheep. *BMC Genomics*, **19**.

72. Miao,X., Luo,Q. and Qin,X. (2015) Genome-wide analysis reveals the differential regulations of mRNAs and miRNAs in Dorset and Small Tail Han sheep muscles. *Gene*, **562**, 188–196.

73. Miao,X., Luo,Q., Qin,X. and Guo,Y. (2016) Genome-wide analysis of microRNAs identifies the lipid metabolism pathway to be a defining factor in adipose tissue from different sheep. *Sci. Rep.*, **5**.

74. Miao,X. and Qin,Q.L.X. (2015) Genome-wide transcriptome analysis of mRNAs and microRNAs in Dorset and Small Tail Han sheep to explore the regulation of fecundity. *Mol. Cell. Endocrinol.*, **402**, 32–42.

75. Sheng,X., Song,X., Yu,Y., Niu,L., Li,S., Li,H., Wei,C., Liu,T., Zhang,L. and Du,L. (2011) Characterization of microRNAs from sheep (Ovis aries) using computational and experimental analyses. *Mol. Biol. Rep.*, **38**, 3161–3171.

76. Wong,L.L., Rademaker,M.T., Saw,E.L., Lew,K.S., Ellmers,L.J., Charles,C.J., Richards,A.M. and Wang,P. (2017) Identification of novel microRNAs in the sheep heart and their regulation in heart failure. *Sci. Rep.*, **7**.

77. Zhao,Q., Kang,Y., Wang,H.-Y., Guan,W.-J., Li,X.-C., Jiang,L., He,X.-H., Pu,Y.-B., Han,J.-L., Ma,Y.-H., *et al.* (2016) Expression profiling and functional characterization of miR-192 throughout sheep skeletal muscle development. *Sci. Rep.*, **6**.

78. Zhou,G., Wang,X., Yuan,C., Kang,D., Xu,X., Zhou,J., Geng,R., Yang,Y., Yang,Z. and Chen,Y. (2017) Integrating miRNA and mRNA Expression Profiling Uncovers miRNAs Underlying Fat Deposition in Sheep. *BioMed Res. Int.*, **2017**, 1–11.
