# Supplementary material for: RumimiR: a detailed microRNA database focused on ruminant species
Source: Database (Oxford). 2019 Oct 14;2019:baz099. doi: 10.1093/database/baz099 (PMC6790497; doi:10.1093/database/baz099)

Python script to compare microRNAs present in RumimiR to small microRNAs extracted from several database.


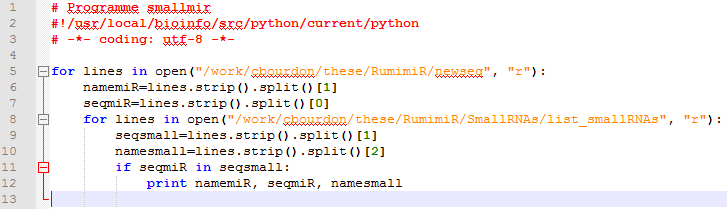


Python script to compare microRNAs present in RumimiR to human or mouse microRNA sequences extracted from miRBase.
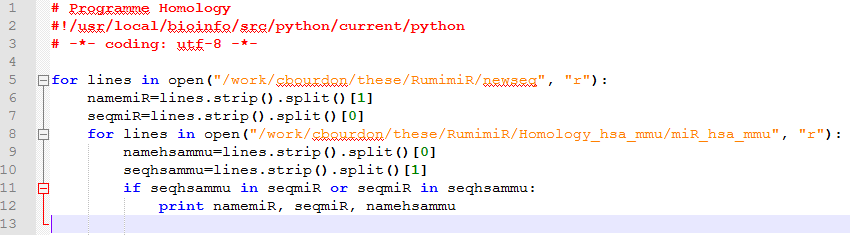

Supplement: Supp_Figure_2_baz099 [file supp_figure_2_baz099.docx]
