# Supplementary figures and images for: RumimiR: a detailed microRNA database focused on ruminant species
Source: Database (Oxford). 2019 Oct 14;2019:baz099. doi: 10.1093/database/baz099 (PMC6790497; doi:10.1093/database/baz099)

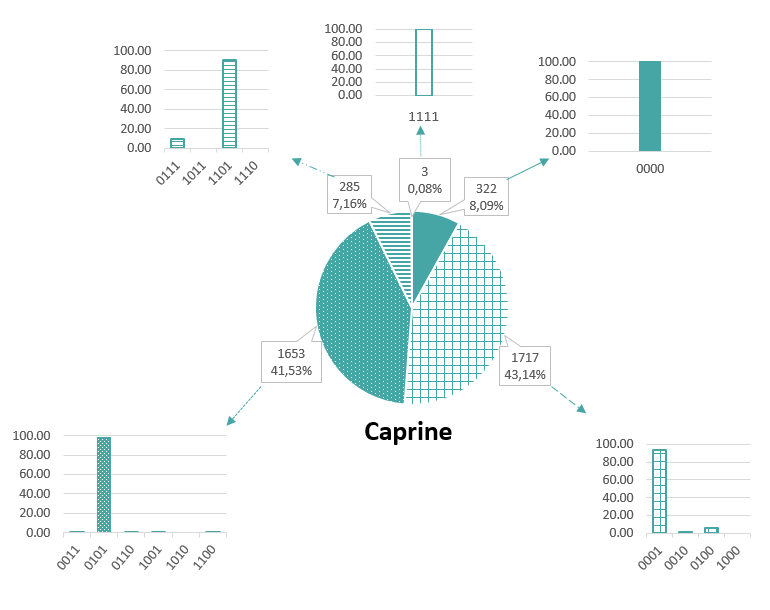

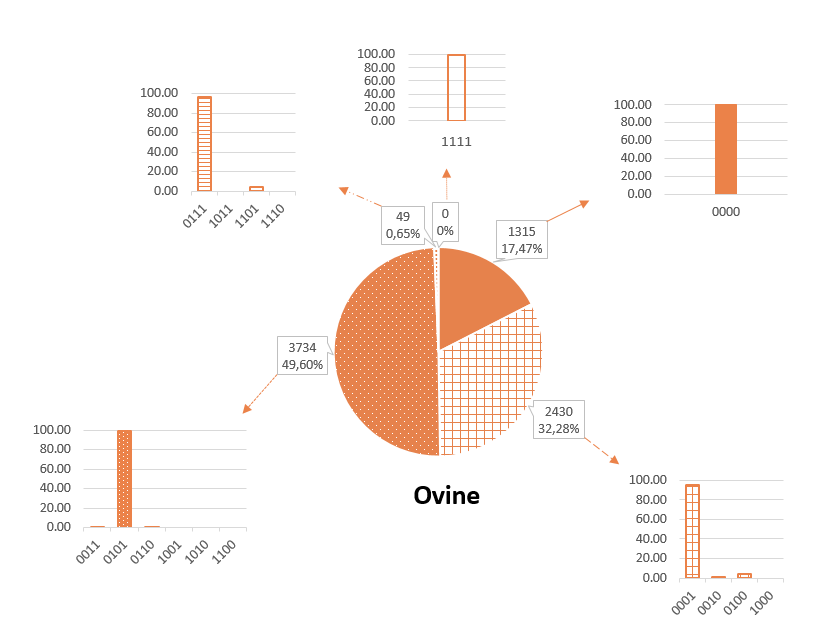

Supplement: Supp_Figure_3_baz099 [file supp_figure_3_baz099.docx]

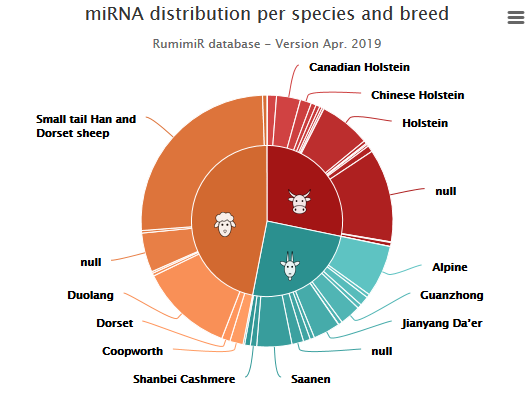


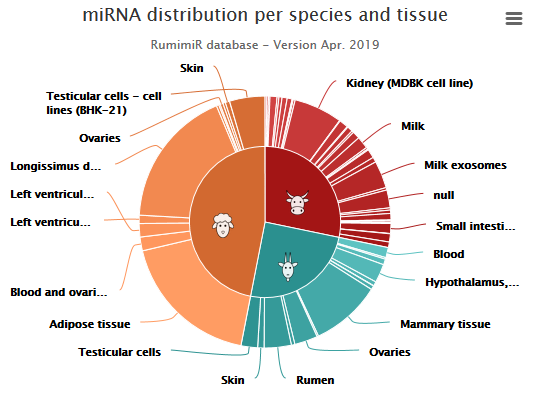


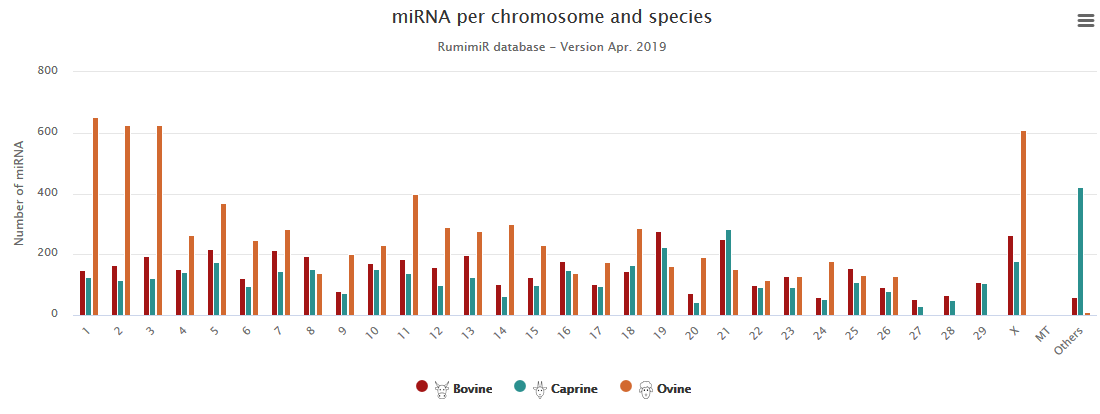


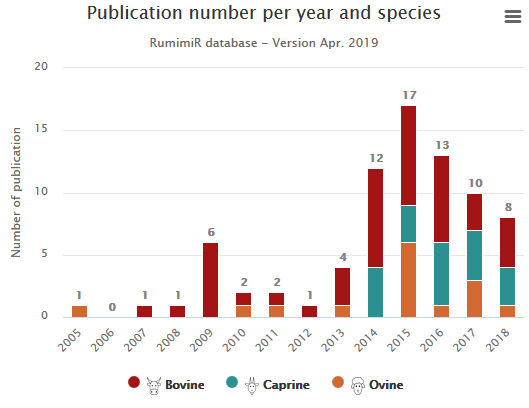


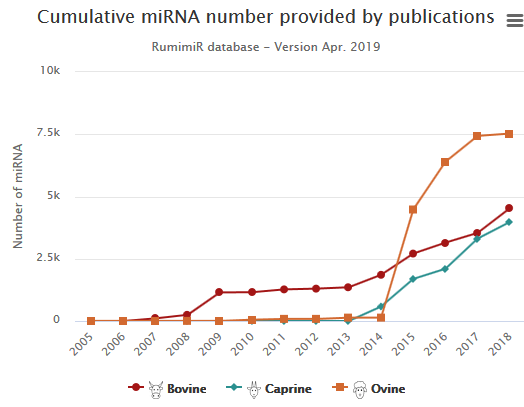

Supplement: Supp_Figure_4_baz099 [file supp_figure_4_baz099.docx]
